# Use of alternative medicine for hypertension in Buikwe and Mukono districts of Uganda: a cross sectional study

**DOI:** 10.1186/1472-6882-13-301

**Published:** 2013-11-04

**Authors:** Fred Nuwaha, Geofrey Musinguzi

**Affiliations:** 1 Department of Disease Control and Environmental Health, School of Public Health, College of Health Sciences, Makerere University, P. O. Box 7072, Kampala, Uganda

**Keywords:** Complementary alternative medicine, Sub-Saharan Africa, Cardiovascular diseases, Non communicable diseases

## Abstract

**Background:**

Use of alternative medicine for chronic diseases such as hypertension is common in low as well as high income countries. This study estimated the proportion of people who were aware of their hypertension that use alternative medicine and identified factors predicting the use of alternative medicine.

**Methods:**

In a community based cross sectional survey among people ≥ 15 years in Buikwe and Mukono districts of Uganda 258 people aware of their hypertension were questioned about use of alternative medicine for hypertension, advice about uptake of life style intervention for hypertension control such as reduction of salt intake and about their attitude towards use of alternative medicine. Proportions of people who used alternative medicine and adopt life style interventions and their 95% confidence intervals (CI) were calculated. Predictors of using alternative medicine were identified using logistic binary regression analysis.

**Results:**

More than a half 144 (56.2%) had ever used alternative medicine whereas more than one in four 74 (28.6%) were currently using alternative medicine alone or in combination with modern medicine (50%). People who were using alternative medicine alone (29.7% CI 17.5-45.9) were less likely to have received advice on reduction of salt intake compared to those using modern medicine alone or in combination with traditional medicine (56.6%, CI 47.7-65.0). The only independent predictor for using alternative medicine was agreeing that alternative medicine is effective for treatment of hypertension (adjusted odds ratio [AOR] 2.6; 95% CI 1.40-4.82).

**Conclusion:**

The use of alternative medicine was common among patients with hypertension and usage was underpinned by the belief that alternative medicine is effective. As patients with hypertension use alternative medicine and modern medicine concurrently, there is need for open communication between health workers and patients regarding use of alternative medicine.

## Background

Alternative, complementary, traditional, unorthodox or unconventional medicine is diverse and difficult to define [1]. In general alternative medicine refers to beliefs and practices not in consonance with ethos of medical community [2,3]. These practices may be well known, mysterious or exotic and some may be dangerous [3]. However, alternative medicine particularly herbs and medicinal plants, are widely used all over the world in high as well as in low income countries [1,4-11]. Reasons for use of alternative medicine may include fear or resentment of modern medicine, ease of access, desire to feel better, being cheap, patients presenting with chronic irritations that are difficult for anyone to treat successfully, curiosity, social influence from friends, relatives and traditional healers as well previous good experience that may be due to placebo or real efficacy [11-17]. Moreover, the use of alternative medicine may be associated with delay in receiving professional care leading to increased complications of disease [11]. Besides, health workers do not take cognizant of the fact that some patients use alternative medicine [1,18-21]. As a result policy and practice regarding use of alternative medicine remain blurred [22,23]. Use of alternative medicine is common in the management of hypertension which is one of the most common non-communicable diseases worldwide affecting up to 20% of the world's adult population [24]. Hypertension is prevalent in many developing countries [24-28] and is the main driver of the cardio vascular disease (CVD) epidemic in Africa where it is a major, independent risk factor for heart failure, stroke and kidney failure [26]. Treatment and control remain sub-optimal; moreover, the health care system is constrained in most of the sub Saharan African countries [25,26]. The use of alternative medicine is increasingly being documented and studied and usage varies between countries with 80% usage reported in Morocco, 48.5% in the Australia, and 40% in the United States [29,30]. The reasons why patients choose to use alternative medicine in the management of hypertension are not fully understood [31].

This study aimed to understand the use of alternative medicine among patients aware that they have hypertension with a view of suggesting measures for improved care. Specifically it estimated the proportion of patients with hypertension that use alternative medicine and analyzed the determinants for use of alternative medicine.

## Methods

### Design

A community cross-sectional survey was carried out in 2012 in Buikwe and Mukono districts of Uganda. The survey that has been described elsewhere [25] measured blood pressure of all people 15 years and above during home visits. In addition people who were aware that they were hypertensive before the survey answered questions regarding treatment and care for their hypertension. Participants were classified as hypertensive aware if they reported that they had previously been informed by a health professional that they had hypertension.

### Setting

Buikwe was carved out of Mukono district in 2009. These two districts lie between the two biggest urban areas of Jinja and Kampala (the capital city). Together the two districts have a population of over 1,000,000 people (projected from the 2002 national census at annual growth rate of 4%). About 80% of the populations are rural dwellers whose main occupation is agriculture. Health services in the two districts are provided by six hospitals, 60 health centres and many private clinics. The major causes of morbidity and mortality are communicable diseases such as malaria, respiratory infections, diarrhoea, HIV/AIDS and tuberculosis. However, non-communicable diseases (NCD) are emerging with hypertension being the commonest reported NCD [32]. Most of the health units are able to diagnose hypertension but drugs for treatment of hypertension are available at the six hospitals and at ten of the health centres which occasionally report drug stock out.

### Sample size

We aimed at interviewing at least 250 respondents aware of their hypertension. This sample size was large enough at 95% confidence interval and a power of 80% with a detectable relative risk of 2 provided that the determinants of using alternative medicine within the study population are not uncommon (less than 20%) or very common (greater than 80%). The sample size assumed that at least a quarter of the respondents use alternative medicine. The sample size was determined using *statcal of EPIINFO* version 6 (http://www.cdc.gov/epiinfo CDC, Georgia) for cross-sectional studies.

### Data collection and measures

A modified questionnaire [11] for health care seeking behaviour was administered to the respondents who reported that they were aware of being hypertensive. Data was collected on treatment and care for hypertension such as type of treatment (modern and or alternative), type of alternative treatment (herbal or otherwise), and life style interventions for hypertension such as advice on salt intake, physical activity, drinking alcohol and on smoking. Among the socio-demographic variables, data was collected on sex, age, place of residence, level of education, marital status and time since diagnosis of hypertension. Furthermore, data was collected on psycho-social factors such as attitude towards alternative medicine, traditional healers, on self treatment and on government health units.

### Analysis

Analysis was done at three levels, univariate, bivariate and multivariable. At univariate level proportion of patients and their 95% confidence intervals (CI) were computed for those that use alternative medicine alone or in combination with modern medicine those that use modern medicine alone and those that neither use modern nor alternative medicine. The proportion of patients that had been advised on life style interventions for hypertension such as reduced salt intake, increased physical activity, reduction or stopping of alcohol consumption and on cessation of smoking were also computed. At bivariate level, social-demographic and psycho-social variables were subjected to a comparison of proportions among users and non users of alternative medicine. For this analysis users of alternative medicine were those using alternative medicine alone or in combination with modern medicine. Non users of traditional medicine were those using modern medicine alone and those that neither use modern nor alternative medicine. At bivariate analysis crude odds ratios (COR) and 95% CI were used as tests of association and significance respectively. All variables that were significant on bivariate analysis (P < 0.05) were entered in a multivariable binary logistic regression model to identify independent predictors of using alternative medicine. Adjusted odds ratios (AOR) and 95% CI were calculated for variables that remained significant after controlling for one another.

### Ethics

The study was approved by Makerere University School of Public Health institutional review board and the Uganda National Council of Science of Technology. Written informed consent was obtained from adult participants. For participants below the age of 18, assent and written informed consent were obtained from minors and their parents/guardians respectively. Patients with uncontrolled hypertension and not on modern treatment were referred to health units.

## Results

Of the 281 patients that were aware of their hypertension 23 (8%) were excluded from further analysis because of missing data. For the remaining 258, 40 (15.5%) were men, 74 (28.7%) were urban dwellers, 189 (73.3%) either had no formal education or had finished primary school, 133 (51.6%) were married, and 167 (57.2%) were less than 55 years of age. Of the sample, 103 (40%) had their hypertension diagnosed within the previous one year, 90 (35%) had their hypertension diagnosed between the last one and four years whereas 65(25%) had their hypertension diagnosed within the past 5 years.

### Treatment for hypertension and use of alternative medicine

As shown in Table 1, 237 (91.8%) had ever used modern medicine and 122 (47.2%) were currently using modern medicine. More than a half 144 (56.2%) had ever used alternative medicine whereas more than one in four 74 (28.6%) were currently using alternative medicine. Of the people currently using alternative medicines, half were only using the remedies for treatment of hypertension. The type of alternative medicine used was almost exclusively herbal remedies reported by 73/74 (99%). A big number of people with hypertension 99 (38.4%) were neither receiving modern nor alternative medicine.

**Table 1:** **Treatment options** (**modern**/**alternative medicine**) **and usage among patients with hypertension**

**Treatment options**	**Number ever used number% (CI)**		**Number of current users number% (CI)**	
Modern medicine alone	103	39.9 (34.1-46.0)	85	32.9 (27.5-38.9)
Modern and alternative medicine (combine)	134	51.9 (46.0-58.2)	37	14.3 (10.6-19.1)
Alternative medicine alone	11	4.3 (2.4-7.5)	37	14.3 (10.6-19.1)
None	10	3.9 (2.1-7.0)	99	38.4 (32.6-44.4)

*CI* 95% confidence interval.

### Life style interventions

Overall 61 (23.6%, CI 18.7-29.1) were advised to increase physical activity, 116 (45.0%, CI 40.1-51.0) were advised to reduce salt intake, 58 (22.5%, CI 17.7-27.8) were advised to reduce or stop use of alcohol and 34 (13.2%, CI 9.4-17.8) were advised to stop smoking. As can be seen in Table 2, use of alternative medicine alone was associated with lower likelihood of being advised to adopt life style interventions particularly for increased physical activity and for reduced salt intake.

**Table 2:** Variations in Life style interventions among patients with hypertension by treatment options for hypertension

	**Treatment option for hypertension**					
**Life style intervention**	**Alternative medicine alone (N** **=** **37)**		**Modern alone or with alternative (N** **=** **122)**		**None neither modern nor alternative (N** **=** **99)**	
	**n**	**% (CI)**	**n**	**% (CI)**	**n**	**% (CI)**
Increase physical activity	4	10.8 (5.1-24.8)	40	32.8 (25.1-41.6)	17	17.2 (11.0-25.8)
Reduce salt intake	11	29.7 (17.5-45.9)	69	56.6 (47.7-65.0)	36	36.4 (27.6-46.2)
Reduce/stop alcohol	7	18.9 (9.6-34.3)	32	26.2 (19.2-34.7)	19	19.2 (12.7-28.1)
Stop smoking	2	5.4 (1.7-17.7)	19	15.6 (10.2-23.1)	13	13.1 (7.9-21.2)

*CI* 95% confidence interval.

### Factors influencing use of alternative medicine

In Table 3 all the socio-demographic variables tested such as year of diagnosis for the hypertension, age of patient, sex, place of residence, level of education and marital status did not influence use of alternative medicine for hypertension (P > 0.05).

**Table 3:** **Crude odds ratios and 95**% **confidence intervals of use of Alternative medicine according to socio**-**demographic characteristics**

**Variable**	Alternative medicine users* (N **=** **74)**	**Alternative medicine non users (N** **=** **184)**	**COR (CI)**	**P-level**
	**n**	**n**		
Year of diagnosis				
< year	26	77	1	
1-4 years	27	63	1.27 (0.64-2.52)	0.56
5 years and above	21	44	1.40 (0.67-2.92)	0.44
Marital status				
Never married	2	5	1	
Currently married	34	99	0.86 (0.14-6.73)	0.99
Divorced or Separated	16	34	1.18 (0.17-9.91)	0.99
Widowed	22	46	1.62 (0.24-13.31)	0.70
Education level				
None	21	45	1	
Primary	32	91	0.75 (0.37-1.53)	0.50
Secondary	16	35	0.98 (0.41-2.31)	0.88
Tertiary	5	13	0.82 (0.22-2.94)	0.97
Age (years)				
< 45	17	59	1	
45 above	57	125	0.64 (0.33-1.25)	0.21
Sex				
Male	10	30	1	
Female	64	154	0.80 (0.37-1.74)	0.70
Place of residence				
Urban	15	59	0.53 (0.28-1.03)	0.08
Rural	59	125	1	

*use alternative medicine alone or in combination with modern medicine.

*COR*: Crude odds ratio; *CI* 95% confidence interval.

Among the psycho-social variables; agreeing that alternative medicine is effective for treatment of hypertension, that traditional healers provide quality treatment for hypertension, are convenient, kind and keep patients secrets, and saying that self treatment for hypertension is good were associated with a higher likelihood of using alternative medicine (P < 0.05). On the other hand, saying that government health units provide quality treatment for hypertension, that free treatment for hypertension is good and that traditional healers delay patients were not associated with use of alternative medicine (P > 0.05) [Table 4]. On multivariable binary logistic regression analysis the only independent predictor for using alternative medicine was agreeing that alternative medicine is effective for hypertension (adjusted odds ratio [AOR] 2.6; 95%CI 1.40-4.82).

**Table 4:** **Crude Odds Ratios and 95**% **confidence intervals of use of Alternative medicine according to psycho**-**social variables**

**Variable**	Alternative medicine users* (N **=** **74)**	**Alternative medicine non users (N** **=** **184)**	**COR (95% CI)**	**P-level**
	**n**	**n**		
Alternative medicine is effective for hypertension				
Agrees	59	53		
Disagrees	15	131	9.72 (5.07-18.63)	< 0.001
Government health units provide quality treatment for hypertension				
Agrees	51	105		
Disagrees	23	79	1.67 (0.94-2.96)	0.11
Free treatment for hypertension is				
Good	70	171	1.33 (0.42-4.22)	0.78
Bad	4	13		
Traditional healers provide quality treatment for hypertension				
Agrees	31	44		
Disagrees	43	140	2.29 (1.29-4.07)	0.006
Traditional healers are convenient for patients with hypertension				
Agrees	40	69		
Disagrees	34	115	1.96 (1.14-3.39)	0.022
Traditional healers are kind to patients with hypertension				
Agrees	40	70		
Disagrees	34	114	1.92 (1.07-3.43)	0.027
Traditional healers keep secrets				
Agrees	27	43		
Disagrees	47	141	1.88 (1.05-3.38)	0.047
Traditional healers delay patients				
Agrees	25	64	0.96 (0.54-1.69)	0.99
Disagrees	49	120		
Self treatment for hypertension is				
Good	42	52	3.33 (1.90-5.83)	< 0.001
Bad	32	132		

*includes those using alternative medicine alone or in combination with modern medicine.

*COR*: Crude odds ratio; *CI* 95% confidence interval.

## Discussion

The use of alternative medicine for hypertension was very high. More than a half of the people aware of their hypertension had ever used alternative medicine whereas more than a quarter were currently using alternative medicine alone or in combination with modern medicine. Among the people with hypertension who use alternative medicine 50% were using it alone without modern medicine. The only independent predictor of using alternative medicine identified in this study was the belief that alternative medicine is effective for the treatment of hypertension. Thus the results of this study complement findings in this setting [11,33] and elsewhere [7-16] that use of alternative medicine is ubiquitous and that its use is underpinned by the common belief that alternative remedies are effective against a number of illness [7,11,12,17,20]. Meanwhile, these results also show that use of modern medicine was nearly universal (92%) and alternative medicine was not used alone but as an adjunct to modern medicine except for only 4% of the subjects who reported using alternative medicine alone. The fact that so few use alternative medicine alone, while a significant proportion use a combination, alternative medicine “on its own” could be perceived as insufficient in the control hypertension among combined users of modern and alternative medicines.

In the current study, psycho-social variables rather than social-demographic variables were the predictors for use of alternative medicine. These data are supported by research from applied social psychology and health education, where attitudinal, social influence and self-efficacy beliefs of this nature are very important in influencing health-related behavior [34,35]. According to social-psychological models, these beliefs are also important in behavior change because they could be targeted for modification using health education/promotion or other appropriate methods [36].

The high rate for concurrent use of alternative and modern medicine found in this study and common elsewhere [7-10] is a cause for concern. Alternative medications may interact with modern medicine causing adverse events [37-39]. Therefore, there is need for health workers to be aware that patients with hypertension use alternative medicine. Because patients do not necessarily volunteer this information [1], health workers should specifically question patients under their care regarding use of alternative medicine. Patients should also be encouraged to volunteer this information to attending clinicians. There is also need to document common herbal remedies used for treatment of hypertension with their possible effects [20] and avail this information to health workers. Collaboration between providers of modern medicine and of alternative medicine as has been reported elsewhere [33] may be a good entry point for this form of documentation.

In the present study patients receiving alternative medicine were less likely to receive life style interventions. Reasons for this observation could be due to selection bias with users of alternative medicines not being comparable to users of modern medicine. Second, it is possible that users of alternative medicine are less likely to consult providers that advise on life style interventions. Third is the possibility that alternative remedies themselves are taken as substitutes for life style interventions by patients as well as traditional healers [33].

This study was aimed at estimating patients with hypertension who use alternative medicine. The strengths of the research design were that patients with hypertension were derived from a community survey rather than health units and the study used a tested protocol that was based on a theoretical methodology [11] for analyzing possible reasons for use of alternative medicine. However, this study had some limitations. First, the psychosocial variables were measured after the behavior (i.e. use of alternative medicine) had occurred. This could have changed their beliefs, particularly the attitudinal ones, depending on the quality of service and the way they were attended to at the places where they received medicines. Second, the cross-sectional nature means that it was difficult to establish cause and effect. For example, it is difficult to conclude whether the belief that alternative medicine is effective against hypertension came before or after using it. Nevertheless, this study provides useful information about use of alternative medicine that may be of practical importance.

## Conclusion

The use of alternative medicine was common among patients aware of their hypertension and usage was underpinned by the belief that alternative medicine is effective. Because patients with hypertension use alternative medicine and modern medicine concurrently, open communication regarding use of alternative medicine is necessary between health workers and patients.

## Competing interests

The authors declare that they have no competing interests.

## Authors’ contributions

Both authors FN and GM took part in conception, design, collection, analysis, interpretation and writing of the manuscript. Both authors approve the final version of manuscript submitted.

## Pre-publication history

The pre-publication history for this paper can be accessed here:

http://www.biomedcentral.com/1472-6882/13/301/prepub

## References

[B1] Eisenberg DM Kessler RC Foster C Norlock FE Calkins DR Delbanco TL Unconventional medicine in the United States - prevalence, costs, and patterns of use N Engl J Med 1993 328 246 252 10.1056/NEJM199301283280406 8418405

[B2] Murray RH Rubel AJ Physicians and healers - unwitting partners in health care N Engl J Med 1992 326 61 64 10.1056/NEJM199201023260113 1727068

[B3] Gevitz N Gevitz N Three perspectives on unorthodos medicine Other healers: Unorthodox medicine in America 1988 Baltimore: Johns Hopkins University Press 1 28

[B4] Cronan TA Kaplan RM Posner L Blumberg E Kozin F Prevalence of use of unconvential remedies for arthritis in a metropolitatin community Arthritis Rheum 1989 32 1604 1605 10.1002/anr.1780321217 2597213

[B5] Verhoef MJ Sutherland LR Brkich L Use of alternative medicine by patients attending a gastroenterology clinc Can Med Assoc J 1990 142 121 125 2295028 PMC1451702

[B6] Clinical Oncology group New Zealand cancer patients and alternative medicine N Z Med J 1987 100 110 113 3470669

[B7] Singh V Raidoo DM Harries CS The prevalence, patterns of usage and people's attitude towards complementary and alternative medicine (CAM) among the Indian community in Chatsworth, South Africa BMC Compl Alternative Med 2004 4 3 10.1186/1472-6882-4-3 PMC356921 15018622

[B8] Eddouks M Maghrani M Lemhadri A Ouahidi ML Jouad H Ethnopharmacological survey of medicinal plants used for the treatment of diabetes mellitus, hypertension and cardiac diseases in the south-east region of Morocco (Tafilalet) J Ethnopharmacol 2002 82 97 103 10.1016/S0378-8741(02)00164-2 12241983

[B9] Delgoda R Ellington C Barrett S Gordon N Younger N The practice of polypharmacy involving herbal and prescription medicines in the treatment of diabetes mellitus, hypertension and gastrointestinal disorders in Jamaica West Indian Med J 2004 53 400 405 15816268

[B10] Shafiq N Gupta M Kumari S Pandhi P Prevalence and pattern of use of complementary and alternative medicine (CAM) in hypertensive patients of a tertiary care center in India Int J Clin Pharmacol Ther 2003 41 294 298 10.5414/CPP41294 12875345

[B11] Nuwaha F Muganzi E Predictors of use of traditional medicine by patients with sexually transmitted infections in Southwest Uganda J Altern Complement Med 2008 14 733 739 10.1089/acm.2007.7160 18684078

[B12] Eisenberg DM Kessler RC Van Rompay MI Kaptchuk TJ Wilkey SA Appel S Perceptions about complementary therapies relative to conventional therapies among adults who use both: results from a National Survey Ann Intern Med 2001 135 344 351 10.7326/0003-4819-135-5-200109040-00011 11529698

[B13] Beecher HK The powerful placebo JAMA 1955 159 1602 1606 10.1001/jama.1955.02960340022006 13271123

[B14] Gillick MR Common-sense models of health and disease N Engl J Med 1985 313 700 703 10.1056/NEJM198509123131120 4022067

[B15] Kronenfeld JJ Wasner C The use of unorthodox therapies and marginal practitioners Soc Sci Med 1982 16 1119 1125 10.1016/0277-9536(82)90114-9 7112162

[B16] Kleinman A Eisenberg L Good B Culture, Illness and care: clinical lessons from anthropologic and cross-cultural research Ann Intern Med 1978 88 251 258 10.7326/0003-4819-88-2-251 626456

[B17] Astin JA Why patients use alternative medicine: results of a national study JAMA 1998 279 1548 1553 10.1001/jama.279.19.1548 9605899

[B18] Ernst E Complementary/alternative medicine for hypertension: a mini-review Wien Med Wochenschr 2005 155 386 391 10.1007/s10354-005-0205-1 16392435

[B19] Yeh GY Davis RB Phillips RS Use of complementary therapies in patients with cardiovascular disease Am J Cardiol 2006 98 673 680 10.1016/j.amjcard.2006.03.051 16923460

[B20] Amira O Okubadejo N Frequency of complementary and alternative medicine utilization in hypertensive patients attending an urban tertiary care centre in Nigeria BMC Compl Alternative Med 2007 7 30 10.1186/1472-6882-7-30 PMC2045097 17903257

[B21] Gohar F Greenfield S Beevers DG Lip G Jolly K Self-care and adherence to medication: a survey in the hypertension outpatient clinic BMC Compl Alternative Med 2008 8 1 10.1186/1472-6882-8-1 PMC2259297 18261219

[B22] World Health Organization WHO traditional medicine strategy 2002–2005 2002 1 Geneva: WHO/EDM/TRM/2002

[B23] Campion EW Why Unconventional Medicine? N Eng J Med 1993 328 282 283 10.1056/NEJM199301283280413 8418412

[B24] Osamor PE Owumi BE Complementary and alternative medicine in the management of hypertension in an urban Nigerian community BMC Complement Altern Med 2010 10 36 1472 6882 10.1186/1472-6882-10-36 PMC2912779 20642829

[B25] Musinguzi G Nuwaha F Prevalence, awareness and control of hypertension in Uganda PLoS ONE 2013 8 4 e62236 10.1371/journal.pone.0062236 23614041 PMC3629133

[B26] Kayima J Wanyenze RK Katamba A Leontsini E Nuwaha F Hypertension awareness, treatment and control in Africa: a systematic review BMC Cardiovasc Disord 2013 13 1 1 11 10.1186/1471-2261-13-1 23915151 PMC3750220

[B27] Gudina EK Michael Y Assegid S Prevalence of hypertension and its risk factors in southwest Ethiopia: a hospital-based cross-sectional survey Integrated blood pressure control 2013 6 111 23986649 10.2147/IBPC.S47298 PMC3753877

[B28] Damasceno A Azevedo A Silva-Matos C Prista A Diogo D Lunet N Hypertension prevalence, awareness, treatment, and control in mozambique Hypertension 2009 54 1 77 83 10.1161/HYPERTENSIONAHA.109.132423 19470872

[B29] Eisenberg DM Trends in alternative medicine use in the United States from 1990-1997 results of a follow-up study JAMA 1998 280 18 1569 1575 10.1001/jama.280.18.1569 9820257

[B30] MacLennan AH Wilson DH Taylor AW Prevalence and cost of alternative medicine in Australia Lancet 1996 347 9001 569 573 10.1016/S0140-6736(96)91271-4 8596318

[B31] Lorenc A Ilan-Clarke Y Robinson N Blair M How parents choose to use CAM: a systematic review of theoretical models BMC Complement Altern Med 2009 9 9 1472 6882 10.1186/1472-6882-9-9 PMC2680396 19386106

[B32] Bbosa RS Caregivers for home based management of fever in Uganda 2010 University of South Africa: Master of Public Health http://uir.unisa.ac.za/bitstream/handle/10500/3649/dissertation_bbosa_r.pdf?sequence=1) Accessed 10 January 2013

[B33] Tumwesigye O Bumetha Rukararwe: integrating modern and traditional health care in southwest Uganda J Altern Complement Med 1996 2 373 10.1089/acm.1996.2.373 9395672

[B34] de Vries H Dijkstra M Kuhlman P Self-efficacy: the third factor besides attitude and subjective norm as predictor of behaviour intentions Health Educ Res 1988 1988 3 273 282

[B35] Ajzen I The theory of planned behaviour Org Beh Hum Dec Proc 1991 1991 50 179 211

[B36] Kok G Schaalma H De Vries H Parcel G Paulussen T Social psychology and health education Eur Rev Social Psychol 1996 1996 7 241 282

[B37] Hughes EF Jacobs BP Berman BM Tierney LM, McPhee SJ, Papadakis MA Complementary and alternative medicine Current Medical Diagnosis and Treatment 2005 New York: McGraw-Hill 1696 1719

[B38] Awang DV Fugh-Berman A Herbal interactions with cardiovascular drugs J Cardiovasc Nurs 2002 16 64 70 10.1097/00005082-200207000-00007 12597263

[B39] Mansoor GA Herbs and alternative therapies in the hypertension clinic Am J Hypertens 2001 14 971 975 10.1016/S0895-7061(01)02172-0 11587167

